# A fast indexing approach for protein structure comparison

**DOI:** 10.1186/1471-2105-11-S1-S46

**Published:** 2010-01-18

**Authors:** Lei Zhang, James Bailey, Arun S Konagurthu, Kotagiri Ramamohanarao

**Affiliations:** 1 National ICT Australia (NICTA) Victoria Research Laboratory at The University of Melbourne, Melbourne, Victoria, Australia; 2 Department of Computer Science and Software Engineering, The University of Melbourne, Melbourne, Victoria 3010, Australia

## Abstract

**Background:**

Protein structure comparison is a fundamental task in structural biology. While the number of known protein structures has grown rapidly over the last decade, searching a large database of protein structures is still relatively slow using existing methods. There is a need for new techniques which can rapidly compare protein structures, whilst maintaining high matching accuracy.

**Results:**

We have developed IR Tableau, a fast protein comparison algorithm, which leverages the *tableau *representation to compare protein tertiary structures. IR tableau compares tableaux using information retrieval style feature indexing techniques. Experimental analysis on the ASTRAL SCOP protein structural domain database demonstrates that IR Tableau achieves two orders of magnitude speedup over the search times of existing methods, while producing search results of comparable accuracy.

**Conclusion:**

We show that it is possible to obtain very significant speedups for the protein structure comparison problem, by employing an information retrieval style approach for indexing proteins. The comparison accuracy achieved is also strong, thus opening the way for large scale processing of very large protein structure databases.

## Background

Protein structure comparison is crucial for understanding protein evolution, architecture and function [1]. The Protein Data Bank (PDB) database [2], a public repository for macromolecule structure data, is a rapidly growing database which currently (as of 4 Aug 2009) contains structural information of 59,330 proteins. The steady growth of the PDB is now beginning to place considerable computational demands on queries which search the entire database, a routine task for determining structural protein similarities. There are a number of existing techniques to compare protein structures: Methods based on structural alignments (for example, DALI [3], SSAP [4] and MUSTANG [5]) compare protein structures at a level of residues (sometime even atoms), and hence detect structural similarities (and differences) with high sensitivity and accuracy. However, the long running times of these methods are prohibitive for exhaustive searches across the entire database. PRIDE [6,7] has been proposed for fast recognition of folds, with reasonable accuracy, using the *C*_*α *_- *C*_*α *_distance profiles of a fixed range of residues. SARST [8-10] utilizes sequence alignment methods to compare Ramachandran codes of different proteins. It is fast enough perform database search. YAKUSA [11] and SHEBA [12] also compare protein structures using their one-dimensional characterizations, either based on protein backbone internal angles or on their environmental profiles. Although these methods are significantly faster than their structural alignment-based counterparts, the lack of global geometric information makes these methods less accurate. Several methods have also been proposed which compare proteins at a coarse level of secondary structures [13-22]. ProSMoS [20] and TableauSearch [15] both try to match the orientation between secondary structure elements (SSEs). Rather than only using angles, OPAAS [13,14,23] uses a probability-based method to align the angle-distance map of SSEs. Mainly, these programs look for similarities in the geometry of interactions between the secondary structural elements in the proteins being compared.

Lesk [24] proposed *tableau *as a concise representation of protein folding patterns. The tableau encodes the geometry of interactions between pairs of secondary structural elements that are in contact [24,25]. Konagurthu et al. [15] proposed three methods to identify structural similarities using a generalized tableau description of protein folding patterns. Their first method allows the identification of identical and near-identical folding patterns in constant time. The second method facilitates a rigorous comparison of two tableaux to identify maximally similar substructures using computationally expensive quadratic and linear integer programming techniques. (We note that Stivala et al. [16] recently gave a faster solution to the quadratic programming formulation of the tableau comparison problem proposed by Konagurthu et al. However, their method still remains infeasible for searching entire databases.) The third method (TableauSearch) was proposed as a fast heuristic to detect similarities using a two-step dynamic programming method.

Most of the existing protein comparison techniques share a major limitation. They are computationally expensive, requiring hours or even days to search a large protein structure database. This has motivated us to develop a new and rapid protein comparison algorithm, IR Tableau, based on feature indexing techniques from Information Retrieval (IR). Our method transforms the robust tableau representation of a protein fold into a vector of *features*, allowing the application of several well-known similarity measures to efficiently compare these feature vectors. IR Tableau achieves excellent search efficiency (it can search the ASTRAL protein structure database for a protein containing 83,731 domains in less than a second), while providing accuracy comparable to the existing methods.

## Methods

This section describes our IR Tableau method, which utilizes IR techniques to index and search protein structures efficiently.

### Tableau representation of protein structure

**Figure 1:**
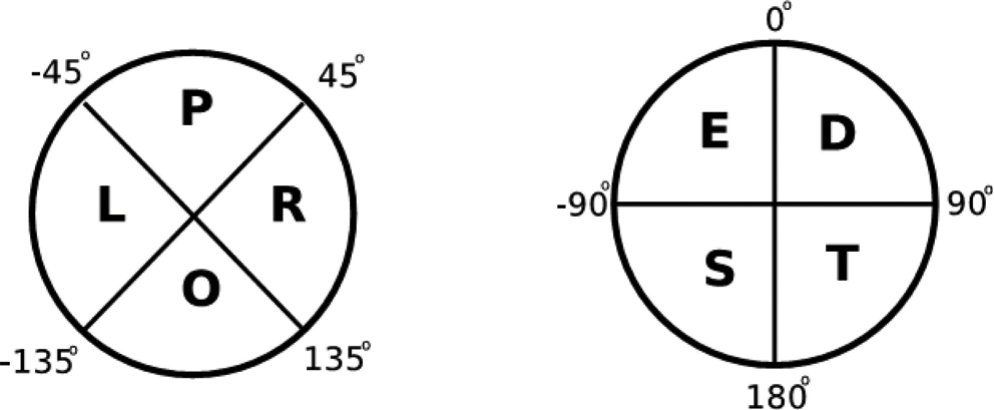
**Tableau orientation encoding scheme**.

Briefly, tableau encodes the geometry of pairs of secondary structural elements (SSEs) - that is, helices and strands of sheet [24]. The relative orientation of a pair of SSEs in a protein is defined by the angle between their axes. Each angle between pairs of SSEs (in the range 180° to 180°) uses a double-character encoding scheme [15]. (See Figure 1.)

There are 8 possible combinations of two characters. For example, Table 1 shows the tableau of a Ubiquitin-like protein, 1UBI (chain A). We used the idea of generalized tableau, which is introduced in [15].

**Table 1:** Tableau representation for 1UBI (chain A). Tableau representation for 1UBI (chain A) containing 6 secondary structure elements. Helix and strand of sheets are represented as *α *and *β *respectively.

SSE	1 *β*	2 *β*	3 *α*	4 *β*	5 *α*	6 *β*
1 *β*	-	OT	LE	RT	RT	PE
2 *β*	OT	-	RT	LE	LS	RT
3 *α*	LE	RT	-	RT	LS	LE
4 *β*	RT	LE	RT	-	PD	OT
5 *α*	RT	LS	LS	PD	-	RT
6 *β*	PE	RT	LE	OT	RT	-

### Information retrieval (IR) approach

A typical IR system aims to retrieve documents that are relevant to keywords (terms) in a user-query. Each document is represented as a vector of weights, where each weight denotes the importance of a given term. Terms are usually the words used by a document and each weight may correspond to the frequency of occurrence of some terms in the document. The collection of all term weights for a document effectively describes the contents of that document. This is known as the 'bag-of-words' model. Different documents can then be compared by comparing their weight vectors. If they use similar weights for each term, then they are likely to be related. Since only vector comparison is used, similarity matching of vectors can be performed extremely fast.

In our protein context, we analogously translate each protein tableau into a vector of weights, where each weight describes the importance of some feature of the protein. Protein structure comparison is then performed by similarity matching of protein vectors. We next describe our technique for creating the vector for a protein structure.

### Protein feature construction method

We generate a vector of features for a given protein based on its tableau representation. So effectively, we translate a two dimensional (2D) tableau into a one dimensional (1D) vector. Each cell in a tableau describes the angle between a pair of SSEs in the protein. For example, in Table 1, the OT in < row 1 column 2> is the orientation between SSE *β *_1 _and SSE *β *_2_. To turn this tableau into a 1D vector, we summarise the distribution of angle frequencies for each possible pair of SSE types.

Each feature of our vector will describe a pair of SSE types in one of eight possible orientations: PE, PD, RD, RT, OT, OS, LS, and LE. The value of each feature corresponds to the frequency at which that configuration occurs in the protein. There are also four possible pairs of SSE types: *αα*, *αβ*, *βα *and *ββ *. Hence each protein can thus be described by 4 *× *8 = 32 features.

Again, in Table 1, there are two *ββ *OT in the tableau of 1UBI, which appear at <row 1, column 2> and <row 4, column 6> in the matrix. Therefore the value for the feature *ββ *OT is 2. The full feature vector for this protein is given in Table 2. In this table, each number indicates the frequency for some combination of SSE types and angle. In summary, there are 32 features, each with an associated frequency count. We construct the above feature vector transformation for every tableau in a structure database. Given a protein structural query, searching can now be performed rapidly in the new 1D feature space.

**Table 2:** Frequency table. Frequency table for combinations of angles and SSE types for the protein 1UBI (chain A). This is information is modelled in our approach using a 32 dimensional vector.

SSE types	PD	RD	RT	OT	OS	LS	LE	PE
*αα*	0	0	0	0	0	1	0	0
*αβ*	0	0	2	0	0	0	1	0
*βα*	1	0	2	0	0	1	1	0
*ββ*	0	0	2	2	0	0	1	1

### Similarity function

Choice of an appropriate similarity function is important for accurate comparison. In IR Tableau, there are a number of possible similarity functions which can be applied for comparing the protein vectors.

*Cosine similarity *[26] simply computes the cosine of the angle between two vectors in a *N *dimensional space. A higher score implies a smaller angle between the two vectors. If the value is 1, it means that the two vectors have the same direction.

The *Jaccard index *[27] is another popular similarity function, defined as the size of the intersection, divided by the size of the union of two sets.

where *A *and *B *are sets.

The *Tanimoto coefficient *[26] is a generalization of the Jaccard index.

*Euclidean distance *is another means of measuring similarity of proteins. Unlike the similarity functions described above, the value for Euclidean distance is not normalized to be between 0 and 1.

where *a*_*i *_is the *i*th element of vector *A*, *b*_*i *_is the *i*th element of vector *B*.

Unless stated otherwise, our results in the rest of the paper assume the use of the cosine similarity function.

### Variation of featuring process

In addition to the method we have described for generating 32 features for each protein (hereafter referred to as the *base method*), we have also explored the value of associating further, additional features with each protein vector. In general, there is a trade-off between adding extra features which can help discriminate between classes of proteins, versus adding too many features which overwhelm accurate similarity calculation.

#### Alternative combinations of SSEs

In our base method, ordering information was only used for pairs of SSE types. This description loses some information about the position of each SSE. By instead preserving positional information about SSEs, we can hope to build a more accurate profile in each protein vector. Incorporating such relationships may be carried out as described in the following example. Protein 1UBI, whose tableau is shown in Table 1 has 6 SSEs: SSE_1 _compared with SSE_2 _is *ββ *OT, SSE_2 _compared with SSE_3 _is *βα *RT and SSE_1 _compared with SSE_3 _is *βα *LE. Combining these, we get the triplet of SSE_1_, SSE_2 _and SSE_3_, which is *ββα *OT RT LE. In general, we can record statistics for all triplets of the form SSE_*m*_, SSE_*m*+1 _and SSE_*m*+2_. (Note that the idea may also be generalized to non-consecutive triplets, such as SSE_*m*_, SSE_*m*+1 _and SSE_*m*+3_). In this triplet approach, there there are 2^3 ^= 8 SSE types and 8^3 ^= 512 angles, giving a space of 8 *× *512 = 4096 possible features. This idea can be further extended to the use quadruplets, quintuplets and so on of SSEs to generate a larger feature space. However, as we can clearly see, the size of the protein vector grows exponentially with the increase in the number of SSEs in each combination. Another possibility is to disregard the ordering information between SSEs, which may be useful for non-linear matching of sub-structures. In this case, there are only three possible orderings, between two SSEs, rather than four: all *αα*, *ββ *and *αβ *. A final possibility is to only consider consecutive SSEs for generating features from a tableau [25]. This can be done by only using the ± 1 off-diagonal entries.

#### Approximate ordering: partitioning the SSE chain

Using the exact order of SSEs as the basis for forming a protein vector can cause the vector to be very large, for complex combinations. To handle this, we have investigated a strategy which uses approximate positions for each SSE, rather than exact positions. Suppose we have a protein with *N *SSEs. The sequence of SSEs can be partitioned into two halves along the chain. All the SSEs in the first-half part will be given a position marker P_1_. SSEs in the second-half will be marked as P_2_. Then, when comparing each pair of SSEs, position markers can be used to provide additional position information. In protein 1UBI, the first SSE compared to the last SSE will be *β *P_1 _*β *P_2 _OT. The number of features generated by using this strategy will then be 4 SSE types *×*8 angles *×*2^2 ^positions = 128 features. If a protein SSE sequence is partitioned into *n *parts, the number of features will be 4 SSE types *× *8 angles *× n*^2^positions.

Datasets

For our experimental evaluation, we use:

1. the entire ASTRAL 1.73 [28] protein domain database. All 97169 protein domains in this data set are processed through the tableau generator program of Konagurthu et al. [24]. The program successfully generated 83,731 tableaux of protein domains covering 1077 different SCOP folds. Using these tableaux, our index database is generated in a single preprocessing step.

2. the ASTRAL 1.73 95% sequence-identity non-redundant data set. We generate our index database from the tableau data set published by Stivala et al. [16] containing 15,169 entries.

We also use the query data set of Stivala et al.'s [16] containing 200 randomly chosen protein domains. Each run using a query returns a list containing all proteins in the respective index databases along with the associated scores.

### Evaluation methodology

All experiments were conducted using a Intel Core 2 Duo 2.4 GHz processor running Ubuntu 9.10 Linux system. IR Tableau was implemented in Java. SCOP [29] fold classification is used as the gold-standard while assessing the accuracy of each search. We use the Receiver Operating Characteristic (ROC) curve, the Area Under this ROC Curve (AUC), Precision-Recall curve and the Mean Average Precision (MAP) to gauge the accuracy.

Given a query protein *P*_*q *_which belongs to the SCOP fold *F*_*q*_, let us consider the top *k *proteins returned by the search as *hits *and the remainder as *misses*. For an *i*th protein  belonging to the SCOP fold , if  = *F*_*q *_and *i *≤ *k *then the protein  is a *true positive *(*TP*). On the other hand, if  ≠ *F*_*q *_and *i *≤ *k *then  is a *false positive *(*FP*). If  ≠ *F*_*q *_and *i *>*k *then  is treated as a *true negative *(*TN*). Otherwise,  is a *false negative *(*FN*). Using the above statistics, we can then compute the true positive rate (*TPR *or recall), false positive rate (*FPR*) and positive predictive value (*PPV *or precision) using the following formulae:

Using the above formulae *TPR*_*k*_, *FRP*_*k *_and *PPV*_*k *_are calculated for all 1 ≤ *k ≤ n*, where *n *is the size of the data set. The ROC defines a curve of points with *FPR*_*k *_as the abscissa and *TPR*_*k *_as the ordinate. Precision-Recall defines a curve with *TPR*_*k *_and *PPV*_*k *_as abscissa and ordinate respectively. For a good search system, the ROC curve is closer to the top-left corner and the Precision-Recall is closer to the top-right corner of the plot.

The area under the ROC curve (AUC) is a single-figure measurement for the quality of an ROC curve. The averaged AUC over our experiment with queries can thus be used to evaluate the method's performance. Mean average precision (MAP) is another useful single-figure accuracy measurement. Here, the average precision over the top *K *positive results is computed by varying *k *over all possible values. The mean of these precisions gives the MAP.

## Results and discussion

In this section we first compare our IR Tableau against several popular methods for protein structure comparison. ProSMoS, OPAAS, SARST and some other web-server based programs are not tested, as results are not comparable. Later, we assess the sensitivity and accuracy of IR Tableau using different types of features defined in this work.

### Comparison between protein comparison algorithms

Table 3 and 4 summarizes the results of comparison of IR Tableau against TableauSearch, Yakusa, QP Tableau, SHEBA, VAST and TOPS. These runs were conducted using 200 queries searching on the two ASTRAL data set. (See Section "Datasets" for more details.)

**Table 3:** Search speed for 200 query set. Results annotated with asterisks ('*') are estimations based on results in [16]. The running time of TableauSearch which was evaluated independently by this work was used to appropriately normalize the results from [16] to account for the differences in machines used between the two works. '--'s indicate that the programs were infeasible to run in a practical time frame. The 'speed-up' columns gives the performance gain as a measure of search speed of IR Tableau with respect to other programs.

Method	Astral 1.73 95%	Speed-up	Astral 1.73 Full	Speed-up
IR Tableau	0.0075 h (27 s)	1×	0.0408 h (147 s)	1×
Yakusa	2.5 h	333×	83 h	2,034×
TableauSearch	0.42 h	56×	3.77 h	92×
QP tableau	222 h*	29,600×	--	--
SHEBA	15.6 h*	2,080×	--	--
VAST	8.4 h*	1,120×	--	--

**Table 4:** AUC results for 200 query set. AUC results for QP Tableau, SHEBA and VAST are taken from [16], which used exactly the same query set and dataset as in our experiments. For IR Tableau, TableauSearch and Yakusa, the output of a query is a list of all proteins along with scores.

Method	AUC
IR Tableau	0.948
Yakusa	0.950
TableauSearch	0.871
QP tableau	0.925
SHEBA	0.941
VAST	0.890
TOPS	0.871

#### Running time performance

Comparing the running times in Table 3, we can clearly see the superior speed of IR Tableau taking merely 27 seconds to complete the 200 query set, while all the other methods take hours and in some cases even days. Searching the full ASTRAL 1.73 database IR tableau takes only 147 seconds, while the search times of other methods increase drastically. We note here that QP Tableau, SHEBA, and VAST are infeasible to run on the full ASTRAL data set. For the smaller ASTRAL 95% data set, the search speeds reported in this work of QP Tableau, SHEBA, VAST, and TOPS are estimations based on the corresponding figures from the work of Stivala et al [16]. These estimations account for the different systems used to conduct our experiments, by using the running time of TableauSearch as a reference to normalize the speeds reported in [16]. (TableauSearch takes 21 minutes in our experiments, while Stivala et al. report 85 minutes for the same job.

More elaborately, on the full ASTRAL 1.73 data set, IR Tableau takes 0.627 seconds per each query. (We note that the comparison time is constant for each pairwise comparison between the query and a database protein.) On the other hand, TableauSearch takes a variable amount of time to complete a query depending on the size of the query. For example it takes 13.38 seconds (CPU time) to search the whole database using SCOP domain d1ubia, a protein which contains only 6 SSEs. But searching on the SCOP domain d1f6dc_ containing 26 SSEs takes 118 seconds. For the same query of d1ubia_, Yakusa takes 26 minutes to search the full ASTRAL 1.73 data set.

#### ROC curve and precision-recall curve performance

**Figure 2:**
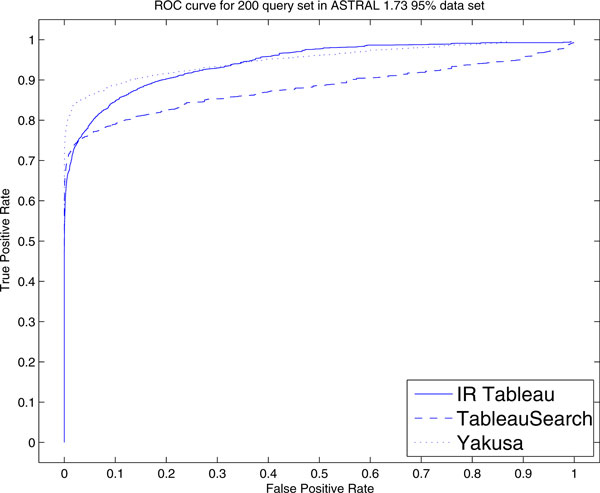
**ROC curves for 200 query set**. ROC curve of IR Tableau, TableauSearch and Yakusa for 200 query set in ASTRAL 1.73 95% data set.

In Table 4, the AUC is shown for the 200 query set. Surprisingly, IR Tableau achieves the second highest AUC value of 0.948. This clearly suggests that the protein feature vectors seem to capture important structural information from tableau. ROC curves for IR Tableau, TableauSearch and Yakusa are shown in Figure 2.

Yakusa has the highest TPR when the FPR is less than 0.35. After this point, IR Tableau becomes slightly better than the other two. TableauSearch is always worse than Yakusa, but better than IR Tableau when the TPR less than 0.3. So in terms of ROC performance, IR Tableau is as good as Yakusa, but over three hundreds times faster.

**Figure 3:**
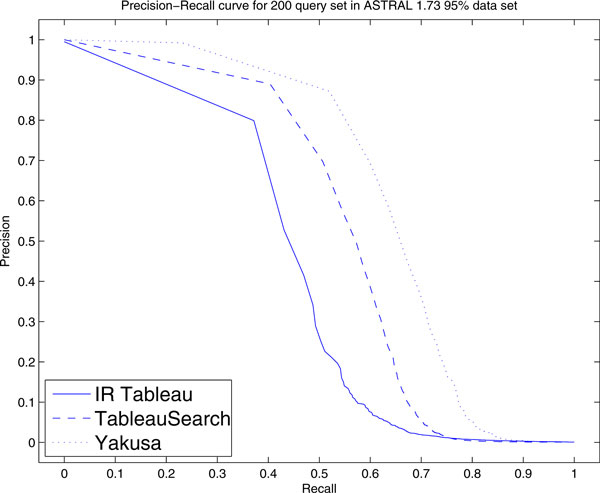
**Precision-recall curves for 200 query set**. Precision-recall curve of i) IR Tableau, ii) TableauSearch and iii) Yakusa for 200 query set in ASTRAL 1.73 95% data set. The MAP scores are respectively i) 0.498, ii) 0.647, iii) 0.74.

The Precision-Recall (PR) curves for IR Tableau, TableauSearch and Yakusa are shown in Figure 3. We note that the performance of both TableauSearch and Yakusa is better than the performance of IR Tableau (their curves are both closer to the upper right corner). Clearly, the Precision-Recall curve exposes differences between the algorithms that weren't apparent in ROC space.

The difference between the behaviour in ROC-space compared to PR-space can be explained based on the imbalance between the classes formed from the top-*k *results when *k *is small. In this circumstance, a small number of positive and a large number of negative results are returned. Therefore a difference in the absolute number of FPs only results in a small change in FPR (as seen in the ROC curves). On the other hand, the same difference in FP results in a large change of precision (as seen in PR curves). In other words, for small *k*, Yakusa and TableauSearch have an advantage in accuracy over IR Tableau, but as *k *becomes larger, all three are very similar.

This suggests that IR Tableau may be very useful to use as a hybrid technique in conjunction with one of these more computationally expensive algorithms. Under this strategy, one would first search the protein database using IR Tableau to return a relatively large set of matches and then pass these results to a second algorithm for deeper, more computationally demanding analysis and reranking of matches.

**Figure 4:**
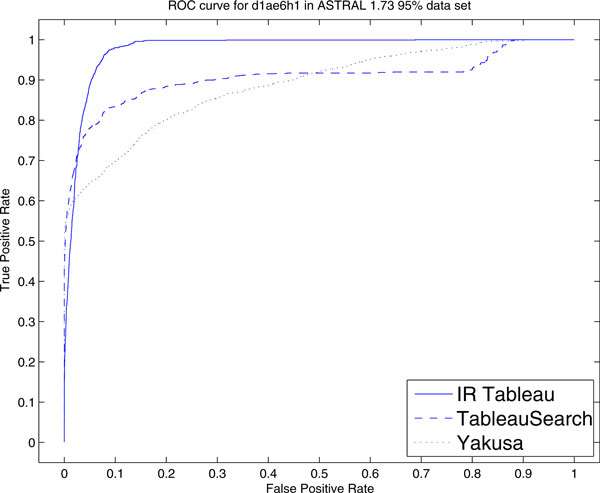
**ROC curve for d1ae6h1**. ROC curve of IR Tableau, TableauSearch and Yakusa for protein d1ae6h1 in ASTRAL 1.73 95% data set.

**Figure 5:**
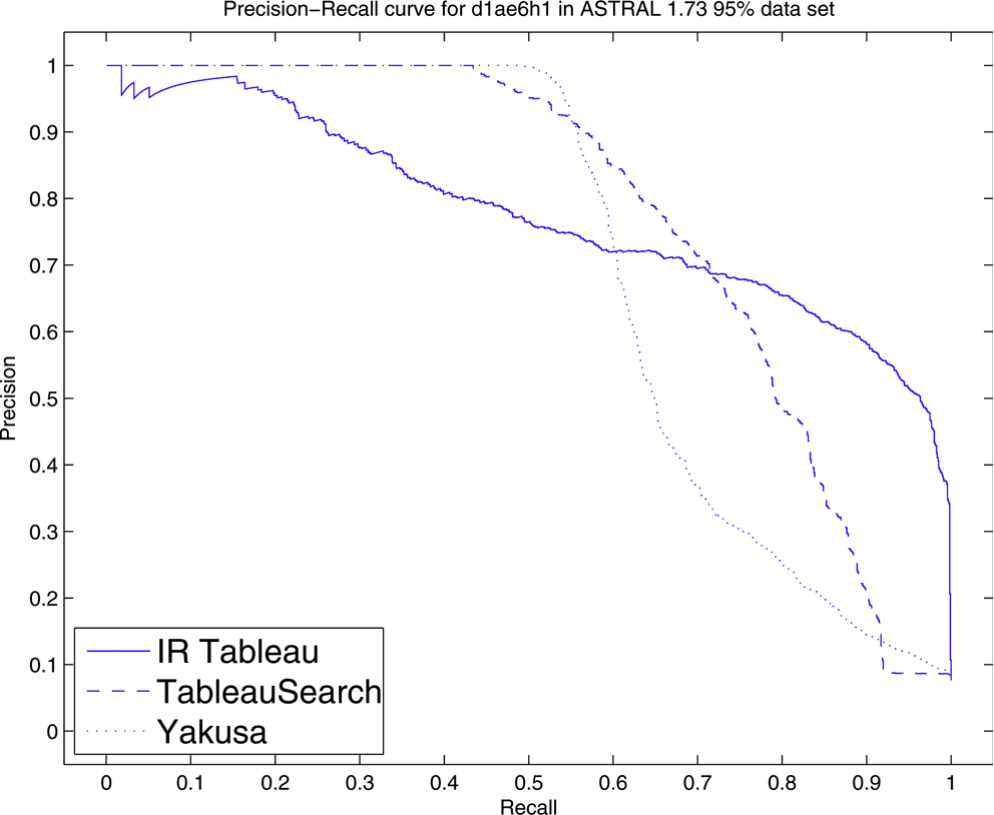
**Precision-recall curve of d1ae6h1**. Precision-recall curve of IR Tableau, TableauSearch and Yakusa for protein d1ae6h1 in ASTRAL 1.73 95% data set.

We also conducted experiments on searching for commonly occurring protein folds. For SCOP domain d1ae6h1, a *β*-grasp protein, the AUC of IR Tableau is 0.978 compared with 0.89 in QP tableau, 0.906 in TableauSearch and 0.887 in Yakusa. This is also better than the version of QP tableau with added SSE distance information, which is 0.95 [16]. The ROC and Precision-Recall curves for this protein search are shown in Figure 4 and 5 respectively.

IR Tableau achieves a superior TPR across almost all the regions in the ROC curve. For the precision-recall curve, the performance of IR Tableau is comparatively not as good for low *k *(low recall), but becomes comparatively better for higher *k *(higher recall). The mean average precision is 0.775 for IR Tableau, 0.777 for TableauSearch and 0.703 for Yakusa.

**Figure 6:**
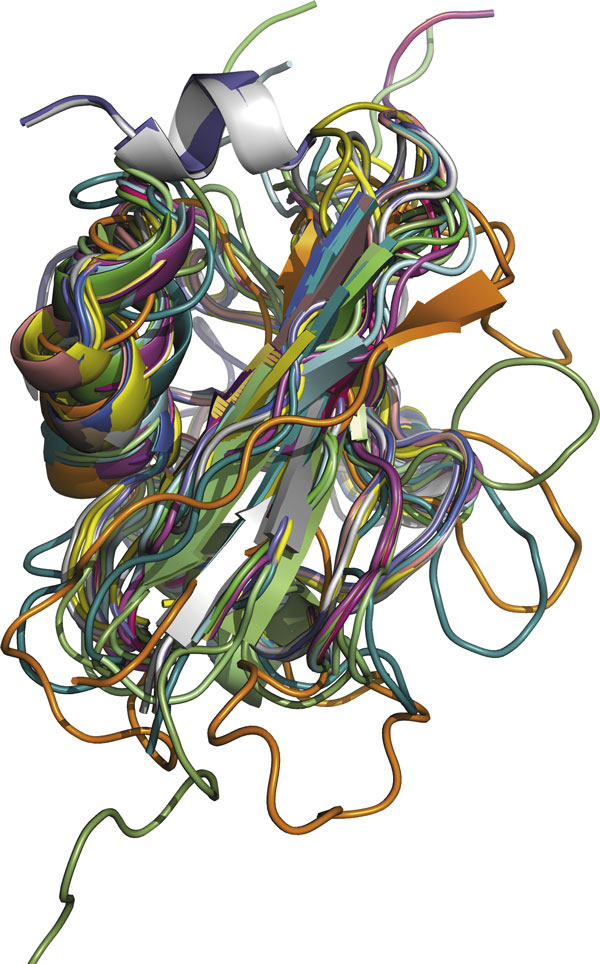
**Superposition graph**. Superposition of the top 20 results using d1ubia as a query protein. MUSTANG [5] was used for structure alignment. This figure is generated using PyMol [30].

In some cases, search results with higher scores are more important than ones with lower scores. Superposition of these returned protein structures is then a very good demonstration of the quality of the top ranked proteins. For protein d1ubia_, the graph in Figure 6 shows the superposition of the top 20 proteins returned by IR Tableau, aligned using MUSTANG [5]. We see that all helices and strands are superposed well, indicating good quality of the results.

### Testing different IR Tableau feature choices

Table 5, shows the results of different featuring methods. If only consecutive SSEs are considered in the indexing process, the AUC and MAP are 0.923 and 0.489 compared with 0.948 and 0.498 for the base method. This difference is relatively small and justifies the notion that consecutive SSEs are important. After discarding all the ordering information between SSEs, the AUC of IR Tableau drops from 0.948 to 0.944, meaning that non-linear structure matching may be easily handled. The accuracy of the triplet approach was very low compared with other featuring methods.

**Table 5:** Behaviour for different featuring methods. Behaviour for different featuring methods using ASTRAL 1.73 95% data set and 200 query set.

Featuring method	AUC	MAP
Base	0.948	0.498
Consecutive SSEs	0.923	0.489
Without ordering	0.944	0.476
Triplet	0.921	0.331

**Figure 7:**
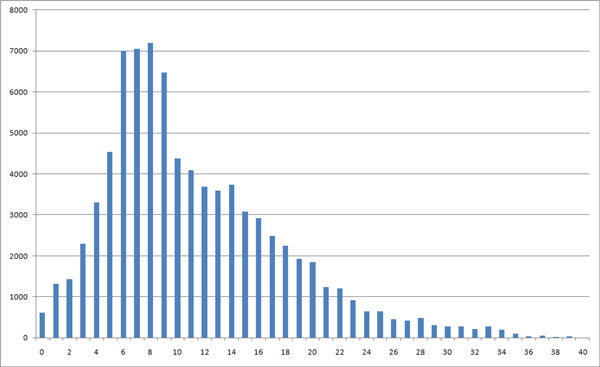
**Distribution of number of SSEs in ASTRAL 1.73 data set**. X axis: number of SSEs in a proteins, Y axis: the number of proteins.

Results of IR Tableau when partitioning proteins into N parts are shown in Table 6. As the number of partitions increases, the AUC gradually decreases. When N is between 5 to 7, the system achieves the highest MAP values. The SSE length distribution in Figure 7 clearly shows that most of proteins have 6 to 8 SSEs.

**Table 6:** Behaviour for different partitioning methods. Behaviour for different partitioning methods using ASTRAL 1.73 95% data set and 200 query set.

No. of Partitions	1	2	3	4	5	6	7	8	9	10
AUC	0.948	0.946	0.940	0.933	0.929	0.918	0.916	0.896	0.892	0.890
Mean Average Precision	0.498	0.537	0.554	0.564	0.573	0.570	0.574	0.568	0.562	0.563

So it is therefore natural that partitioning the SSE chain into 5 to 7 parts works well, since it means that we are effectively trying to match the position of each SSE exactly.

The behaviour for different similarity functions is shown in Table 7. Cosine similarity has the highest AUC 0.948, followed by the Tanimoto coefficient at 0.947. Even the worst, Jaccard index, still achieves 0.906. With respect to MAP, the differences are greater. The Tanimoto coefficient and Jaccard index have very low MAP, while Euclidean distance gives the highest MAP. Unlike the other measures, Euclidean distance is not normalized. If two proteins greatly differ in size, we can expect un-normalized similarity measures will reduce the distance greatly.

**Table 7:** Behaviour for different similarity functions using ASTRAL 1.73 95% data set and 200 query set

Similarity function	AUC	MAP
Cosine similarity	0.948	0.498
The Jaccard index	0.906	0.347
The Tanimoto coefficient	0.947	0.244
Euclidean distance	0.932	0.544

## Conclusion

We have introduced IR Tableau, a new algorithm for protein structure comparison. A key advantage is that it is highly scalable, being faster than existing methods, by over a factor of 100. This speed up factor also increases for longer proteins. Moreover, it is able to achieve good quality of search results, obtaining comparable AUC scores to existing algorithms and slightly lower MAP scores. Highly efficient search algorithms will be very important for protein structure databases of the future, which may contain millions of proteins. We believe that our IR Tableau approach is very promising for such a scenario. In particular, it may be used as part of a hybrid filter approach. The user can run IR Tableau for a high throughput scan of the database for approximate matches. The search results would then be passed to a second algorithm, for deeper (and more computationally expensive) comparative analysis. Conducting experiments along these lines is an interesting avenue for future work.

## Competing interests

The authors declare that they have no competing interests.

## Authors' contributions

All authors contributed to this paper equally.
